# Cholesterol Levels Are Not Associated with Peripheral Blood Stem Cell Mobilization in Healthy Donors

**DOI:** 10.3390/jcm14176239

**Published:** 2025-09-04

**Authors:** Sema Seçilmiş, Burcu Aslan Candır, Ersin Bozan, Samet Yaman, Bahar Uncu Ulu, Tuğçe Nur Yiğenoğlu, Dicle İskender, Merih Kızıl Çakar, Mehmet Sinan Dal, Fevzi Altuntaş

**Affiliations:** 1 Department of Hematology and Bone Marrow Transplantation Center, Ankara Oncology Training and Research Hospital, Ankara 06200, Turkey; drburcuaslancandir@gmail.com (B.A.C.); ersinbozan87@gmail.com (E.B.); drsametyaman@hotmail.com (S.Y.); baharuncu@gmail.com (B.U.U.); dr.nuryigenoglu@gmail.com (T.N.Y.); diclekoca@yahoo.com (D.İ.); merihkizil@yahoo.com (M.K.Ç.); dr.sinandal@gmail.com (M.S.D.); faltuntas@hotmail.com (F.A.); 2 Department of Internal Medicine & Hematology, School of Medicine, Yıldırım Beyazıt University, Ankara 06760, Turkey

**Keywords:** cholesterol, hematopoietic stem cell mobilization, CD34^+^ cells, healthy donors, peripheral blood stem cells

## Abstract

**Background/Objectives:** Hematopoietic stem cell (HSCs) mobilization from the bone marrow to the peripheral blood (PB) is a critical step in stem cell transplantation. Although some experimental studies have suggested that cholesterol levels may affect this process, the clinical relevance of lipid profiles in healthy donors remains unclear. This study aimed to investigate whether serum cholesterol parameters are associated with peripheral blood CD34+ HSC mobilization in healthy stem cell donors. **Methods:** A total of 251 healthy donors who underwent granulocyte colony-stimulating factor (G-CSF)-based mobilization were retrospectively analyzed. Peripheral blood CD34+ cell counts and yields (×106/kg) were recorded. Laboratory parameters, including total cholesterol, HDL-C, LDL-C, and triglyceride levels were evaluated. Correlations between mobilization outcomes and donor characteristics or laboratory findings were also assessed. **Results:** No significant association was found between serum lipid parameters (total cholesterol, LDL-C, HDL-C, triglycerides) and CD34+ cell mobilization or yield. However, white blood cell count, hemoglobin level, platelet count, absolute neutrophil count, and lymphocyte count showed significant positive associations with mobilization efficacy. In contrast, body mass index (BMI) was inversely correlated with CD34+ cell yield. **Conclusions:** Serum cholesterol levels do not appear to influence stem cell mobilization outcomes in healthy donors. Classical hematologic parameters remain reliable predictors of CD34+ cell yield. These findings suggest that cholesterol is not a suitable biomarker for predicting mobilization efficiency in this population group.

## 1. Introduction

Peripheral blood stem cell (PBSC) mobilization is a cornerstone of allogeneic hematopoietic stem cell transplantation (HSCT), enabling the collection of hematopoietic stem cells (HSCs) for therapy. Mobilization is most commonly achieved through the administration of granulocyte colony-stimulating factor (G-CSF), which promotes the release of HSCs into the peripheral blood for subsequent collection via leukapheresis. However, the response to G-CSF varies significantly among donors, and predicting mobilization efficiency remains a clinical challenge. Donor-specific variables, such as age, sex, body mass index (BMI), and baseline hematologic parameters, have been identified as predictors of mobilization success [1,2].

Recently, metabolic factors, particularly cholesterol, have emerged as potential modulators of hematopoiesis. Cholesterol is a vital component of cell membranes that contributes to structural integrity, protein function, and intracellular signaling. It also serves as a precursor for steroid hormones and bile acids and plays a role in maintaining cellular and systemic homeostasis [3,4,5].

In the context of hematopoiesis, cholesterol may influence HSC behavior through various mechanisms. It modulates endothelial cell function and interacts with the bone marrow microenvironment. Preclinical studies have demonstrated that elevated cholesterol levels can disrupt the stromal cell-derived factor 1 (SDF-1)/C-X-C chemokine receptor type 4 (CXCR4) axis, which regulates stem cell retention in the bone marrow, leading to enhanced HSC mobilization into the peripheral circulation [6]. Although a limited number of retrospective studies have examined the impact of cholesterol levels on stem cell mobilization in patient populations [7,8,9], to our knowledge, no large-scale studies have evaluated this association exclusively in healthy donors [10]. Previous studies have demonstrated that classical hematological parameters, such as white blood cell (WBC) count and platelet levels, are consistently associated with mobilization efficiency [1,2]. In addition to cholesterol, other metabolic and inflammatory factors, such as triglycerides, hormones, and cytokines secreted by adipose tissue, have been increasingly implicated in the regulation of hematopoietic activity and immune cell trafficking. These components interact with bone marrow stromal cells and may influence stem cell niche function, although their precise roles in PBSC mobilization remain unclear [5,11]. While some clinical studies have reported a positive association between elevated serum cholesterol levels and improved PBSC mobilization [8,12], others have found no such relationship [9], indicating conflicting evidence across different populations and mobilization protocols. In contrast to studies involving patient populations, which are often affected by comorbidities and treatment-related variables, our investigation in a healthy donor cohort provides a clearer understanding of the intrinsic donor-related determinants of mobilization capacity.

Identifying reliable predictors of mobilization success is crucial for donor safety, ensuring efficient stem cell collection, reducing the need for additional apheresis sessions, and minimizing procedure-related complications.

In this study, we aimed to determine whether cholesterol levels are predictive factors for PBSC mobilization in healthy individuals. Additionally, we assessed other clinical and hematologic variables that may influence the CD34+ cell yield and circulating CD34+ cell counts. To achieve this, we conducted a retrospective analysis of 251 healthy donors who underwent mobilization with only G-CSF.

## 2. Materials and Methods

This retrospective study was approved by the Ethics Committee of Ankara Dr. Abdurrahman Yurtaslan Oncology Training and Research Hospital (approval number 2022-02/1650, dated 9 February 2022) and was conducted in accordance with the Declaration of Helsinki and institutional ethical standards.

### 2.1. Study Design and Participants

A total of two hundred and fifty-one healthy adult donors (≥18 years) who underwent PBSC mobilization for allogeneic transplantation between January 2010 and April 2021 were included. The donors were HLA-matched and selected based on institutional and international eligibility criteria. Informed consent was obtained for G-CSF administration and apheresis.

All participants underwent a comprehensive pre-donation evaluation, including physical examination, electrocardiography, and radiography. Individuals with chronic illnesses, those receiving lipid-lowering treatment, and active smokers were excluded based on previous studies suggesting negative associations between these conditions and CD34+ cell counts [13,14]. Blood samples were collected after an overnight fast of at least 12 h.

### 2.2. Mobilization and Collection Procedure

The donors received subcutaneous recombinant G-CSF (filgrastim) at 10 µg/kg/day for four consecutive days. On day five, peripheral blood CD34+ cell counts were measured using multicolor flow cytometry, and stem cell collection was initiated approximately two hours after the final G-CSF dose. G-CSF administration continued until either the target CD34+ dose of 4 × 106 cells/kg was reached [15] or mobilization failure was identified, defined as the failure to collect at least 2 × 106 CD34+ cells/kg of the recipient’s body weight [16].

Apheresis was performed using a continuous flow cell separator. A maximum of three sessions was allowed based on yield. CD34+ cell doses were expressed as ×106/kg relative to both recipient and donor body weights to account for the interindividual variability.

### 2.3. Laboratory Measurements

Prior to the first apheresis session, venous blood was drawn to assess the lipid profiles, including total cholesterol (T-Chol), LDL cholesterol (LDL-C), HDL cholesterol (HDL-C), and triglycerides (TGs). Additional laboratory parameters included hemoglobin (Hb) level, platelet count, white blood cell (WBC) count, absolute neutrophil count (ANC), and lymphocyte count.

### 2.4. Outcome Measures

The primary endpoints were the peripheral blood CD34+ cell count (cells/μL) on day 5 and the total CD34+ cell yield obtained during apheresis, recorded as ×106 per kilogram of donor weight.

## 3. Statistical Analyses

All statistical analyses were performed using IBM SPSS Statistics for Windows, Version 25.0 (IBM Corp., Armonk, NY, USA). Descriptive statistics were expressed as frequencies and percentages for categorical variables, and as mean ± standard deviation for continuous variables. The Kolmogorov–Smirnov test was used to assess the normality of distributions; all variables showed *p* > 0.05 and were thus considered normally distributed. Pearson correlation analysis was applied to evaluate the relationships between continuous variables. A *p*-value < 0.05 was considered statistically significant. To visually demonstrate the associations between cholesterol levels and CD34^+^ cell counts, scatter plots with linear regression trend lines were generated.

## 4. Results

This study included 251 healthy donors. The mean age was 40.6 ± 13.5 years, and 39.4% of participants were female. Donors were classified into four groups according to the World Health Organization body mass index criteria (underweight (BMI < 18.5 kg/m^2^), normal (18.5 kg/m^2^ ≤ BMI < 25 kg/m^2^), overweight (25 kg/m^2^ ≤ BMI < 30 kg/m^2^), and obese (BMI ≥ 30 kg/m^2^) [17]. The comprehensive demographic characteristics and baseline laboratory values are presented in Table 1.

On the fifth day of G-CSF administration, the mean peripheral blood stem cell count was 70.5 ± 33.3 cells/µL. No donor in this cohort experienced mobilization failure in this study. The target CD34+ cell dose was obtained after a single apheresis session in 140 (55.8%) donors. Two procedures were required in 102 individuals (40.6%), whereas only nine donors (3.6%) underwent a third session. The additional procedural outcomes are summarized in Table 2.

**Figure 1:**
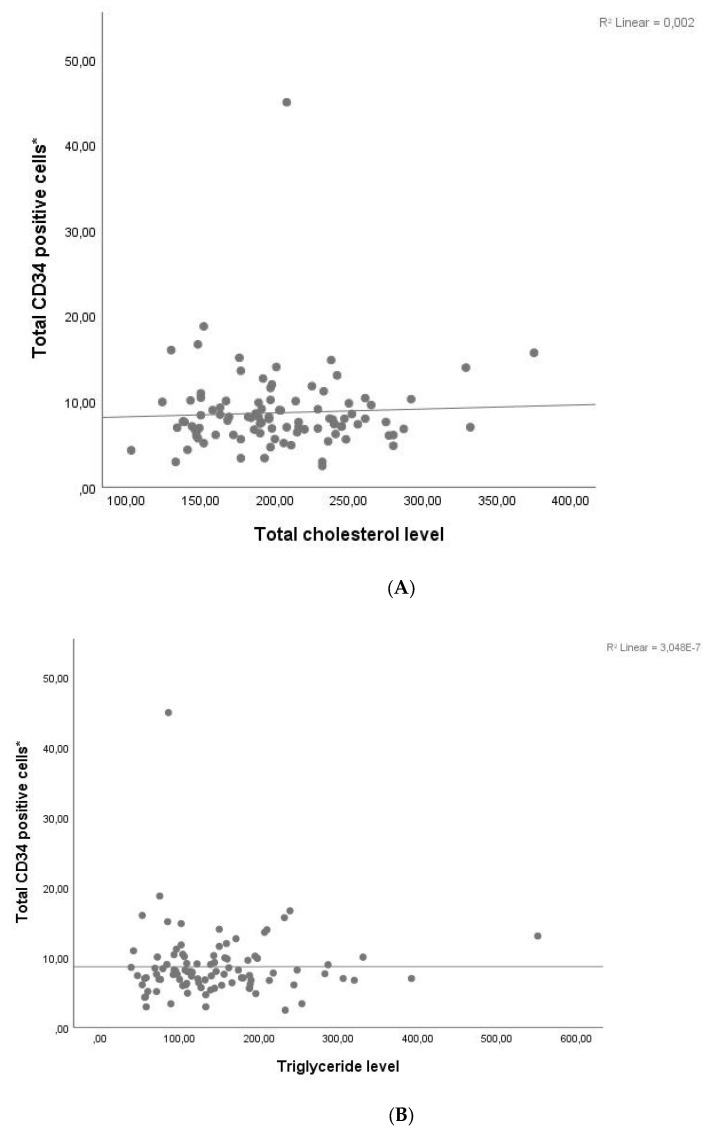

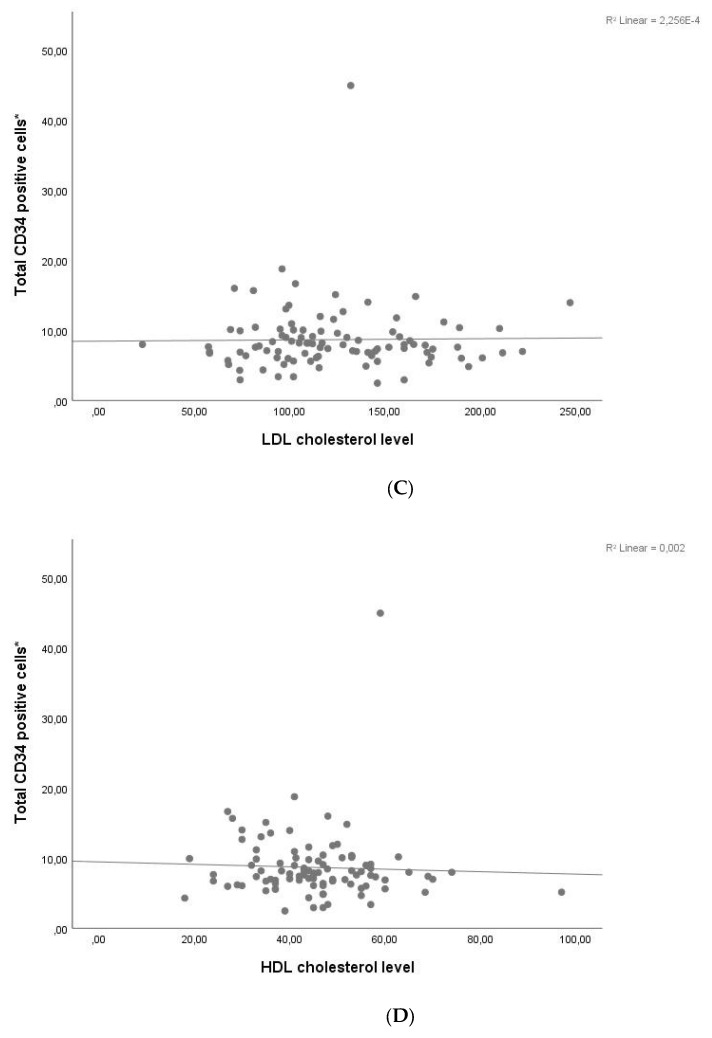
Scatter plots illustrating the relationship between the CD34^+^ HSC yield (×10^6^/kg of donor weight) and donor lipid parameters: (**A**) total cholesterol (T-Chol) (r = –0.023, *p* = 0.716), (**B**) triglycerides (TG) (r = –0.108, *p* = 0.088), (**C**) low-density lipoprotein cholesterol (LDL-C) (r = 0.000, *p* = 0.996). (**D**) high-density lipoprotein cholesterol (HDL-C) (r = 0.019, *p* = 0.769). Each dot represents an individual donor. None of the correlations were statistically significant. A linear trend line is shown in each plot. *; CD34 positive cells ×10^6^/kg of donor weight.

This study also examined the clinical and laboratory variables associated with peripheral blood CD34+ cell counts. As presented in Table 3, a significant positive correlation was observed between the PBSC count and several hematologic parameters, including the white blood cell, hemoglobin, platelet, absolute neutrophil, and lymphocyte counts. Additionally, the CD34+ cell dose collected during both the initial and cumulative apheresis sessions (expressed as ×106/kg of recipient body weight) was significantly correlated with PBSC count. In contrast, no significant associations were found between donor age, body weight, height, BMI, monocyte count, or total cholesterol, triglyceride, HDL-C, or LDL-C levels. As illustrated in Figure 1, no significant correlations were observed between peripheral blood CD34+ cell counts and donor lipid parameters, including total cholesterol, triglyceride, HDL-C, and LDL-C levels.

**Figure 2:**
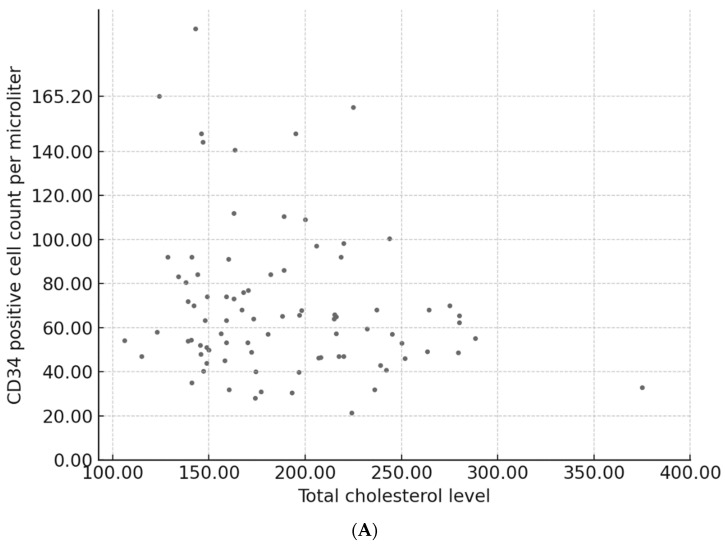

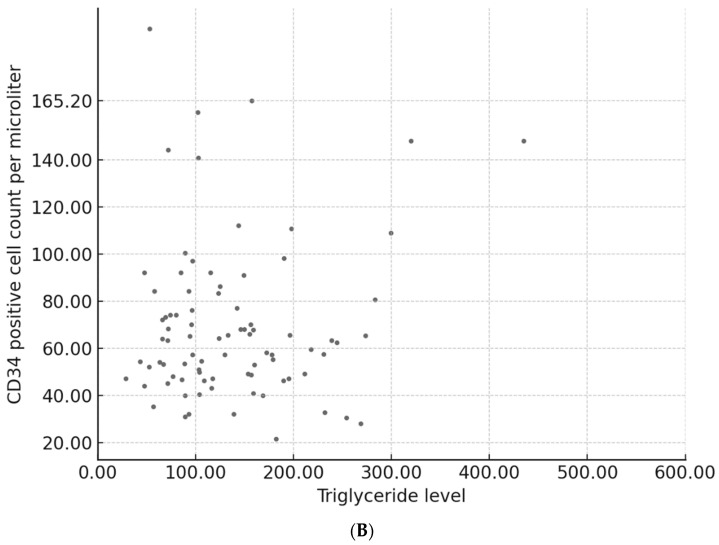

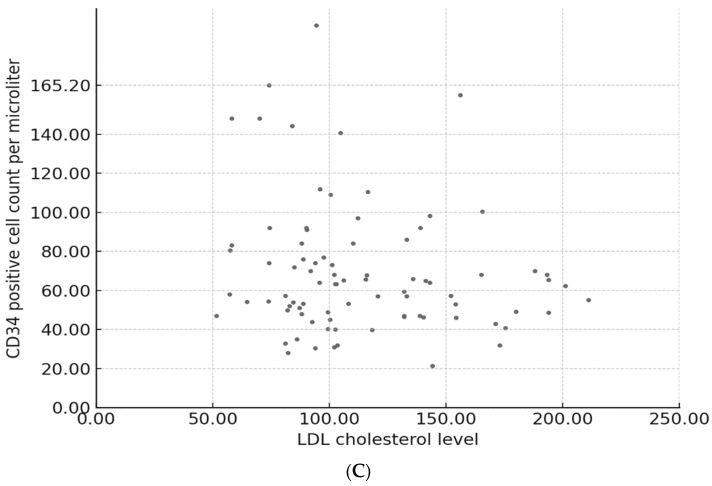

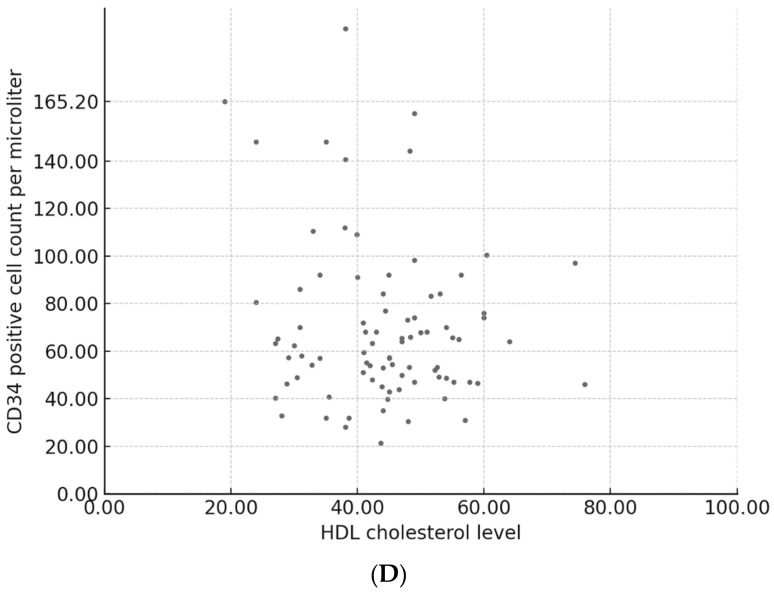
Scatter plots illustrating the relationship between peripheral blood CD34+ hematopoietic stem cell (HSC) counts and donor lipid parameters: (**A**) total cholesterol (T-Chol) (r = –0.055, *p* = 0.390), (**B**) triglycerides (TG) (r = 0.060, *p* = 0.347), (**C**) low-density lipoprotein cholesterol (LDL-C) (r = –0.029, *p* = 0.646). (**D**) high-density lipoprotein cholesterol (HDL-C) (r = –0.005, *p* = 0.932). Each dot represents an individual donor. No significant correlations were observed in any of the groups. A linear regression trend line is shown in each panel.

We also assessed the association between the CD34+ cell yield per kilogram of donor body weight and various clinical parameters. As summarized in Table 4, the CD34+ cell yield was inversely correlated with donor height, body weight, BMI, and hemoglobin levels. In contrast, a strong positive correlation was observed between CD34+ cell yield and peripheral blood CD34+ cell count, as well as between the initial and cumulative apheresis-derived CD34+ cell doses (expressed as ×106/kg recipient weight). No statistically significant associations were found between CD34+ cell yield and donor age, pre-apheresis WBC count, platelet count, ANC, lymphocyte count, monocyte count, or lipid profile parameters, including total cholesterol, triglyceride, HDL-C, and LDL-C levels. Figure 2 shows that the CD34+ HSC yield per kilogram of donor weight was not significantly associated with any of the lipid parameters.

## 5. Discussion

This study obtained the target CD34+ hematopoietic stem cell count after the first apheresis in 140 donors (55.8%), which is compatible with previous reports indicating success rates of 63–92% [18,19]. In our study, we did not observe any mobilization failures. Previous studies have determined that poor mobilization occurs in 2–5% of healthy donors [1]. In our study, we excluded donors with incomplete file data from the analysis, as some of these donors may have experienced mobilization failure.

We found no significant relationship between serum cholesterol levels and peripheral blood CD34+ HSC counts. Mobilization is known to depend on the disruption of the stromal cell-derived factor 1 (SDF-1)/C-X-C motif chemokine receptor 4 (CXCR4) axis, particularly under the effect of G-CSF or CXCR4 antagonists. Research in mouse models has demonstrated that high cholesterol levels, particularly high LDL cholesterol levels, can increase SDF-1 levels in the peripheral blood. This increase disrupts the SDF-1:CXCR4 axis within the bone marrow, allowing the mobilization of lymphocytes, neutrophils, platelets, and hematopoietic progenitor cells from the bone marrow [6]. However, this mechanism has been described in murine models, not in healthy human donors.

Previous clinical studies exploring cholesterol and PBSC mobilization have reported conflicting results. A study involving 82 patients who underwent autologous transplantation reported higher PBSC counts in individuals with elevated total cholesterol levels [8], whereas another study involving 52 patients observed no significant correlation [9]. Importantly, these studies involved patient populations with comorbidities that can influence cholesterol metabolism, cytokine levels, and G-CSF response.

A study involving 7216 healthy donors found that female sex, older age, and smoking were negatively correlated with CD34+ cell counts. Conversely, a higher platelet count, absolute lymphocyte count, relative monocyte count, and baseline BMI were positively correlated with the CD34+ cell count [1]. Another study reported a positive association between CD34+ cell count and variables such as platelet count, hemoglobin level, white blood cell count, body mass index, and triglyceride levels, while also revealing a negative correlation with HDL cholesterol and the degree of height loss [20]. Our study focused on healthy donors, thus providing a clearer picture of donor-intrinsic variables. We identified significant positive correlations between the PBSC count and the baseline WBC, hemoglobin, platelet, absolute neutrophil count, and lymphocyte counts. These parameters may reflect a more robust bone marrow reserve and responsiveness to G-CSF stimulation. However, factors such as donor age, weight, height, BMI, and monocyte count did not have a significant impact on PBSC count.

The quantity of CD34+ cells in pre-apheresis blood is linked to a greater yield of CD34+ cells and is a strong predictor of successful PBSC collection [21]. Our findings are also consistent with previous studies [1,21], indicating that the yield of CD34+ cells is related to the PB CD34+ cell count and the first and total apheresis CD34+ cell doses (x106/kg of the recipient). Furthermore, we observed that the CD34+ cell yield per kg was negatively associated with donor height, weight, BMI, and hemoglobin levels.

Numerous studies have shown that BMI significantly affects peripheral blood progenitor cell yield [1,22,23]. This positive effect may be partly explained by the relatively higher doses of G-CSF administered to donors with an elevated BMI. Interestingly, recent animal studies have demonstrated that adipose tissue, owing to its structural similarity to bone marrow, may also participate in hematopoietic activity by supporting stem cell populations [24].

Although BMI was strongly correlated with G-CSF-induced HSC mobilization, our study found a significant negative correlation between the yield of CD34+ cells and BMI. Excessive adipose tissue may eventually affect stem cell mobilization in various ways by altering the marrow environment and promoting inflammatory cytokine activity. Therefore, the relationship between BMI and PBSC yield remains uncertain, and additional studies are necessary to clarify the role of BMI in G-CSF-induced stem cell mobilization.

Age is a significant predictor of successful PBSC collection. In a retrospective study involving 175 donors, the factors associated with the yield of CD34+ cells included age, baseline platelet levels, and pre-collection hematopoietic progenitor cells [25]. In contrast, in our study, similar to the study by Tabilio et al. [26], no correlation was found between donor age and CD34 yield. This may be due to environmental exposure and genetic predisposition, which could act as additional determinants of CD34+ cell yield in donors.

Biologically, cholesterol plays a multifaceted role in hematopoiesis. It is integral to membrane fluidity, lipid raft formation and receptor signaling. Increased cholesterol levels promote HSC proliferation and mobilization. There is increasing evidence that cholesterol homeostasis is an important factor in the regulation of hematopoiesis [5]. However, plasma cholesterol levels may not reflect cellular cholesterol activity accurately, as systemic levels depend on complex exchanges among organs and tissues [3].

From a clinical perspective, identifying reliable predictors of mobilization success is critical for ensuring donor safety and operational efficiency. Predictive markers can help tailor mobilization regimens, avoid unnecessary G-CSF exposure or additional apheresis sessions, and ensure adequate graft quality in the future. While our results do not support the use of cholesterol as a useful standalone marker in healthy donors, traditional hematologic parameters remain relevant.

This study had several limitations. This was a retrospective, single-center analysis without longitudinal follow-up. We did not include a comparison group of hyperlipidemic donors under treatment, which may have further clarified the cholesterol–mobilization relationship. Furthermore, we relied on serum lipid values at baseline, without functional cholesterol assays or cytokine profiling.

Nevertheless, our findings are important as they address a gap in the literature by evaluating the role of cholesterol in stem cell mobilization in a relatively large cohort of healthy donors. Future prospective studies should explore the integration of metabolic, genetic, and inflammatory markers to improve the predictive accuracy of HSC mobilization.

## 6. Conclusions

In this study involving 251 healthy donors, we found no significant association between serum cholesterol levels and the mobilization of peripheral blood CD34+ hematopoietic stem cells. In contrast, classical hematologic parameters, including white blood cell count, hemoglobin level, platelet count, and absolute neutrophil count, were more strongly correlated with mobilization efficiency. Although body mass index showed an inverse association with CD34+ cell yield, the role of lipid metabolism in this process remains unclear.

Our findings suggest that serum cholesterol should not be considered a predictive marker of PBSC mobilization in healthy individuals. Nevertheless, these results may contribute to optimizing donor selection strategies and mobilization protocols in the future. Further prospective studies are warranted to elucidate the underlying molecular, inflammatory, and metabolic mechanisms that regulate hematopoietic stem cell mobilization.

## Figures and Tables

**Table 1:** Data on demographic characteristics and laboratory parameters of the donors.

Variables	Total
Patient weight (kg)	71.4 ± 13.3
Donor gender, *n* (%)	
Male	152 (60.6)
Female	99 (39.4)
Donor age (year)	40.6 ± 13.5
Donor height (cm)	168.7 ± 9.0
Donor weight (kg)	76.8 ± 14.9
Donor BMI (kg/m^2^), *n* (%)	27.0 ± 4.8
Underweight	13 (5.2)
Normal	77 (30.7)
Overweight	92 (36.7)
Obese	69 (27.4)
Pre-leukapheresis blood counts	
WBC (×10^3^/µL)	42.0 ± 13.2
ANC (×10^3^/µL)	35.0 ± 12.4
Hb (g/dL)	14.3 ± 1.4
Plt (×10^3^/µL)	233.5 ± 61.7
Lymphocyte (×10^3^/µL)	3.7 ± 1.6
Monocyte (×10^3^/µL)	2.4 ± 1.4
T-chol (mg/dL)	193.2 ± 45.7
TG (mg/dL)	147.3 ± 118.4
HDL-C (mg/dL)	45.5 ± 10.8
LDL-C (mg/dL)	117.8 ± 38.1

Continuous variables were presented as mean ± standard deviation (Mean ± SD). Body mass index was calculated as weight divided by height squared (kg/m^2^).

**Table 2:** Apheresis products.

Variables	Values
PBSC count/µL	70.5 ± 33.3
CD34+ HSCs *collected in the first apheresis	6.2 ± 2.8
Total CD34^+^ HSC * collected	8.0 ± 3.4
CD34^+^ HSC ** yield	7.6 ± 3.8
Number of donor apheresis procedures, *n* (%)	
∎ 1 session	140 (55.8)
∎ 2 sessions	102 (40.6)
∎ 3 sessions	9 (3.6)

PBSC: Peripheral blood stem cell; HSCs: Hematopoietic stem cells; *: ×10^6^/kg of recipient weight; **: ×10^6^/kg of donor weight; Continuous variables were presented as mean ± standard deviation (Mean ± SD).

**Table 3:** The relationship between PB CD34+ HSC count/µL and clinical variables.

Variables	r	*p*
Donor age	−0.106	0.096
Donor height	0.053	0.413
Donor weight	−0.005	0.935
Donor BMI	−0.041	0.528
Pre-leukapheresis blood counts		
WBC	0.283 **	<0.001
Hb	0.142 *	0.025
Plt	0.133 *	0.036
ANC	0.278 **	<0.001
Lymphocyte	0.132 *	0.038
Monocyte	0.096	0.133
T-chol	−0.055	0.39
TG	0.06	0.347
HDL-C	−0.005	0.932
LDL-C	−0.029	0.646
In the first apheresis, collected CD34^+^ HSCs (×10^6^/kg recipient)	0.450 **	<0.001
Total CD34^+^ cell count (×10^6^/kg recipient)	0.141 *	0.026
The yield of CD34^+^ cells (×10^6^/kg donor)	0.029	0.652

* Correlation is significant at the 0.05 level (Pearson correlation test). ** Correlation is significant at the 0.01 level (Pearson’s correlation test). r = Pearson’s correlation coefficient; *p* = *p*-value for significance.

**Table 4:** The relationship between clinical variables and the yield of CD34^+^ cells (×10^6^/kg of the donor weight).

Variables	*r*	*p*
Donor age	−0.019	0.763
Donor height	−0.206 **	0.001
Donor weight	−0.308 **	<0.001
Donor BMI	−0.231 **	<0.001
Pre-leukapheresis blood counts		
WBC	0.038	0.552
Hb	−0.188 **	0.003
Plt	0.006	0.925
ANC	0.072	0.257
Lymphocyte	0.015	0.809
Monocyte	−0.066	0.300
PB CD34^+^ cell count/µL	0.146 *	0.040
In the first apheresis, CD34^+^ cells (×10^6^/kg recipient)	0.332 **	<0.001
T-chol	−0.023	0.716
TG	−0.108	0.088
HDL-C	0.019	0.769
LDL-C	0.000	0.996
Total CD34^+^ cell count (×10^6^/kg recipient)	0.865 **	<0.001

PB: Peripheral blood; * Correlation is significant at the 0.05 level (Pearson correlation test). ** Correlation is significant at the 0.01 level (Pearson’s correlation test). r = Pearson’s correlation coefficient; *p* = *p*-value for significance.

## Data Availability

Data used in this study are available upon request.
